# Lead Sequestration from Halide Perovskite Solar Cells with a Low-Cost Thiol-Containing Encapsulant

**DOI:** 10.1021/acsami.2c05074

**Published:** 2022-06-23

**Authors:** Rene D. Mendez L., Barry N. Breen, David Cahen

**Affiliations:** ± Department of Chemistry and Nanotechnology and Advanced Materials Center, Bar Ilan University, Ramat Gan 52900, Israel; § 3GSolar Photovoltaics Ltd., Jerusalem 9777403, Israel; ° Weizmann Institute of Science, Rehovot 76100, Israel

**Keywords:** perovskite, lead, leaching, encapsulation, sequestration

## Abstract

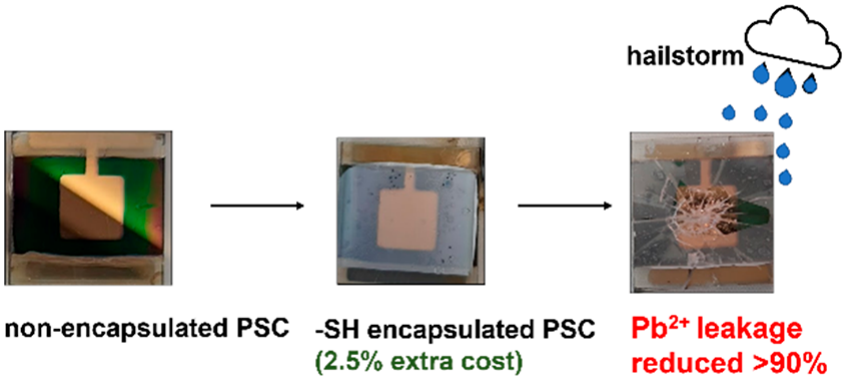

Perovskite solar cells (PSCs) are being studied and developed because of the outstanding properties of halide perovskites as photovoltaic materials and high conversion efficiencies achieved with the best PSCs. However, leaching out of lead (Pb) ions into the environment presents potential public health risks. We show that thiol-functionalized nanoparticles provide an economic way of minimizing Pb leaching in the case of PSC module damage and subsequent water exposure (at most, ∼2.5% of today’s crystal silicon solar panel production cost per square meter). Using commercial materials and methods, we retain ∼90% of Pb without degrading the photovoltaic performance of the cells, compared with nonencapsulated devices, yielding a worst-case scenario of top-soil pollution below natural Pb levels and well below the U.S. Environmental Protection Agency limits.

## Introduction

In the past decade, halide perovskites (HaPs) have been researched intensively as absorber materials for photovoltaic (PV) devices (as well as light-emitting and radiation/particle detection materials). Such PV cells have shown tremendous advance in terms of the power conversion efficiency (PCE), the rate at which solar light can be converted into electrical energy, starting from 3.8% in 2009 to 25.5% in 2021 for a single-junction device.^1^

Perovskite solar cells (PSCs) with the best PV performance are based on lead (Pb) halide frameworks. Upon exposure to water, the Pb compounds that form, lead dihalides, are sparingly soluble in water. Importantly, solar cells are macroelectronic devices, meaning that commercially large areas of the cells are needed. The large areas imply that, even with low solubility, Pb that can leach from damaged cells into the environment, soil, or groundwater after contact with moisture cannot be ignored. Water, either in the liquid or vapor state, can travel through HaP films (∼0.6 μm thick) along grain boundaries and quickly (<3 min) start to dissolve the material, visibly changing the perovskite from a dark-black opaque film to a yellow transparent film.^2^

PbI_2_ is the main Pb-containing decomposition product in the most efficient solar cells and thus is likely the main product to leak from broken solar modules. The PbI_2_ solubility in water is 756 mg L^–1^ (at 20 °C),^3^ while by chemical laboratory standards, this counts as “poorly soluble”, the maximum accepted levels of Pb in drinking water are set as 5 orders of magnitude lower, at 15 μg L^–1^ (15 ppb) by the U.S. Environmental Protection Agency (EPA).^4^ These numbers demonstrate the importance of being able to control and limit the possible escape of dissolved Pb-containing products from PSCs into the environment because these can be carried away with water to the soil below and onward to water flows that are or reach water reservoirs, potentially harming life that depends on that water.

An accident scenario has been assessed^2^ in which HaP is completely exposed to simulated rain of pH values ranging from 4.2 to 8.1, causing conversion to PbI_2_, which is dissolved. These experiments, which were done with CH_3_NH_3_PbI_3_ samples, showed that all of the Pb content would be leached out in a span of 5 min to 1 h.

The amount of Pb in a PSC with a 600-nm-thick HaP absorber layer is 0.82 g m^–2^. For the experiments carried out in this work, we use devices with 3.36 cm^2^ area, which translates into 275 μg of Pb in each device. If all of the Pb from a single PSC with this exposed area leached into water, it could potentially pollute up to 18 L of water, above the EPA^5^ Pb-permissible levels in drinking water. This means that a 1 m^2^ area of PSC can pollute up to 54 m^3^ of *stagnant* water to a level >15 ppb.

A strategy to mitigate Pb water pollution from a PSC is through the use of encapsulants, as reported already for self-healing polymers, which can reduce Pb leakage by more than 2 orders of magnitude compared with those based on UV-cured resins;^6^ another example is the use of transparent phosphonic acid films on a cover glass, which can prevent Pb leaching from encapsulated PSCs by up to 96% compared to nonencapsulated ones.^7,8^ Other reports note that cation-exchange resin (CER) can collect >90% of the Pb from encapsulated modules,^9,10^ while further research publications discuss Pb recycling from PSCs into new devices with fair performance.^11−13^

Previously, other reports have suggested retarded Pb leakage during the initial 10 min of water exposure from PSC with hole-transport materials containing a thiol (SH) group.^14−17^ Because Pb ions are known to have a strong affinity for sulfur,^18−20^ we propose an alternative encapsulant material with Pb-sequestering properties based on a common, silicone-based adhesive in combination with SH groups from (3-mercaptopropyl)trimethoxysilane (MPTMS)-capped nanospheres (MPTMS-ns). The cost of the sequestrant materials is ∼ US$1.1 m^–2^ [see the Supporting Information (SI) for calculations], which represents about 2.5% of today’s (2021) current silicon (c-Si) solar panel manufacturing cost ($43/m^2^).^21^ Because the volume of PSC production (>1 GW year^–1^), which may be achieved by 2025, has to be competitive with that of c-Si panels, a similar ∼2.5% added cost can be expected for them. Because the MPTMS-ns fraction of the *cost* of the panels is calculated from the commercial, laboratory-scale *price*, a large-scale purchase (which is possible because it is already produced in bulk quantities by chemical companies) will likely further reduce this fraction of the cost (estimated at ∼0.07 US$ m^–2^)^22^ Moreover, our Pb sequestrant does not decrease the PV output of a PSC while capturing an average of 90% of the leaching Pb ions from broken devices.

## Results and Discussion

### MPTMS-ns Trapping of up to 98% Pb in Water

**Figure 1:**
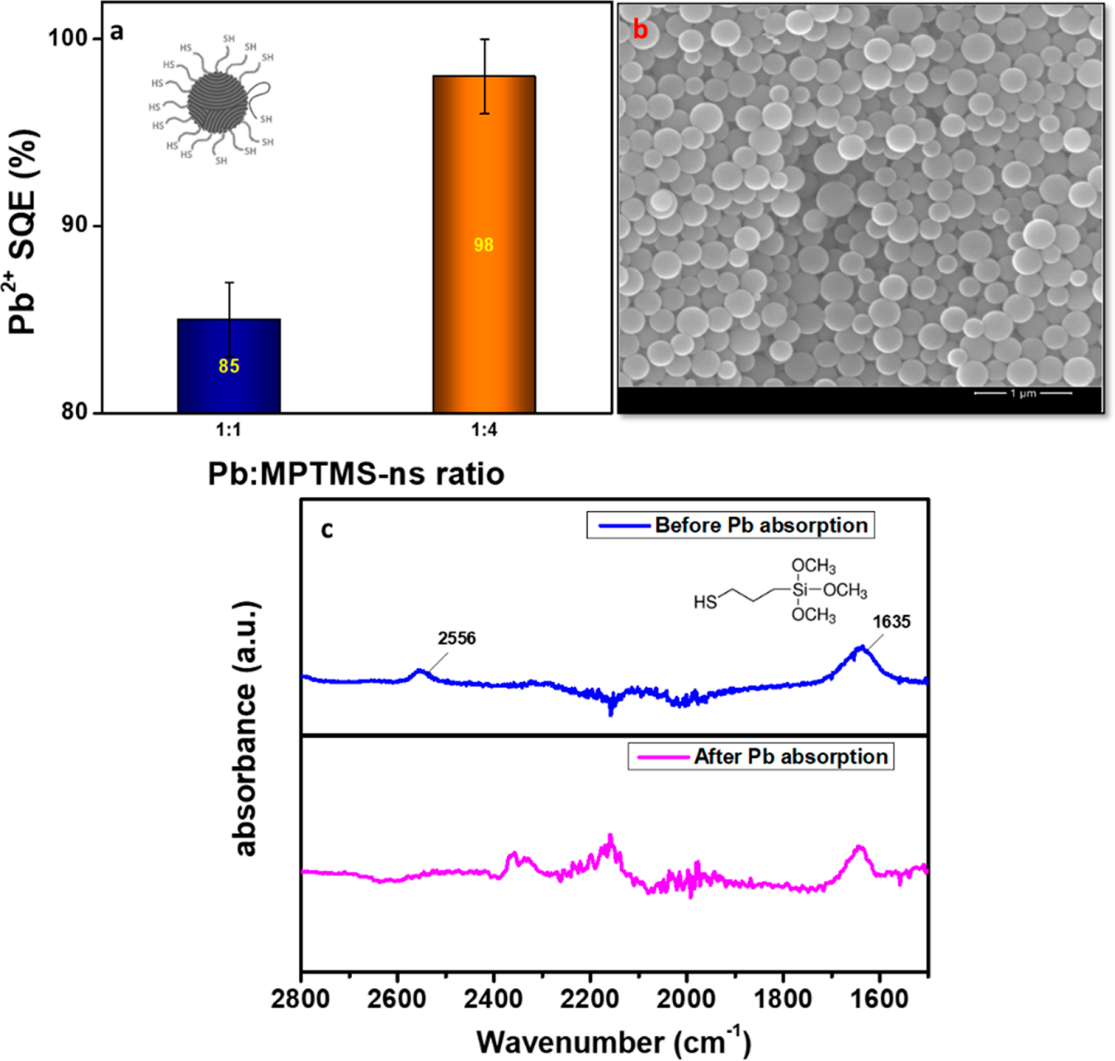
MPTMS-ns characterization and Pb SQE. (a) Pb^2+^ SQE (%) from polluted water with decomposition products from HaP layers, by adding MPTMS-ns (inset illustration) at two different weight ratios. (b) SEM micrograph of MPTMS-ns, synthesized by sol–gel preparation. The average sphere diameter is ∼200 nm. (c) FTIR spectra of the MPTMS-ns material before (blue) and after (pink) exposure to Pb^2+^ ions, with the precursor MPTMS molecule drawn as an inset.

MPTMS-ns were synthesized using a procedure from the literature;^23^ the spherical silica particles are sufficiently functionalized so as to provide SH group loading on the nanosphere surface, showing a strong S–H bond stretching vibration with a peak at 2556 cm^–1^ in the Fourier transform infrared (FTIR) spectrum (Figure 1c). The peak at 2556 cm^–1^, which is absent after the absorption of Pb^2+^, is consistent with the S–H groups binding with Pb^2+^ by electrostatic interactions. The peak at 1635 cm^–1^ is related to hydrogen bonds from water (O–H stretch). MPTMS-ns are small enough, on average ∼200 nm in diameter (Figure 1b), to be integrated within a sealant and provide Pb sequestration properties. The percentage of Pb collected from HaP layers *with* MPTMS-ns absorbers in comparison with Pb collected in HaP layers *without* absorbers is defined as the Pb sequestration efficiency (SQE). To evaluate the Pb SQE for our MPTMS-ns, we submerged individual HaP layers of 6.45 cm^2^ (1 in^2^) area in 15 mL of water and measured an average Pb concentration of 35 ppm with flame atomic absorption spectroscopy (FAAS). Then, we added two different w/w ratios of 1:1 and 1:4 (Pb:MPTMS-ns) to the polluted water. The Pb SQE values were above 85% and 98%, respectively (Figure 1a).

### MPTMS-ns Compatibility with a Silicone-Based Encapsulant for PSCs

The criteria for choosing a proper adhesive for sealing the PSC and integrating the addition of MPTMS-ns for Pb sequestration in the device were as follows: the adhesive must be nonreactive to HaPs or any of its constituents, compatible as a host for MPTMS-ns (silica core nanoparticles), able to flow before curing, moisture-resistant, and thermally curable. We found that the Dow silicone-based adhesive satisfied the above criteria because it was used successfully in industry for dye-sensitized solar cells that contain iodide in a liquid organic electrolyte, with no degradation of the adhesive by iodide exposure.^24^

### Pb Trapping (90%) and Leakage Rate with Integrated MPTMS-ns Encapsulation in PSCs

**Figure 2:**
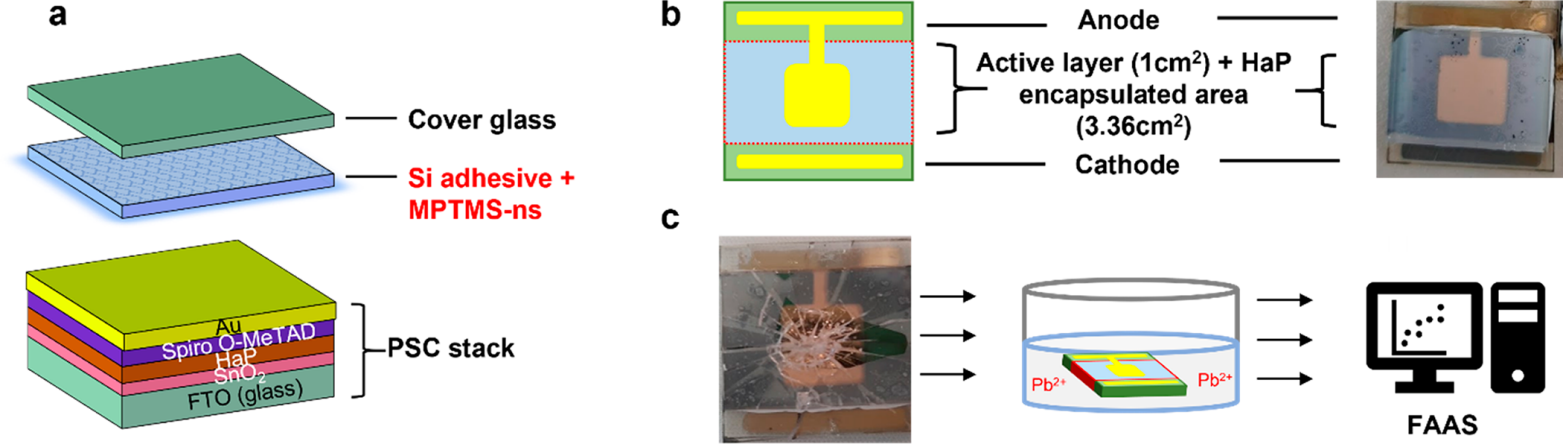
PSC encapsulation and Pb SQE methodology. (a) Illustration of MPTMS-ns–silicone encapsulant location within a n–i–p PSC. (b) Top-view scheme and photograph of an encapsulated PSC with a total HaP area of 3.36 cm^2^. (c) Experimental procedure: breaking the PSC, dipping the broken cell in 15 mL of water for 24 h, and elemental analysis by FAAS to determine the amount of leached Pb^2+^.

**Figure 3:**
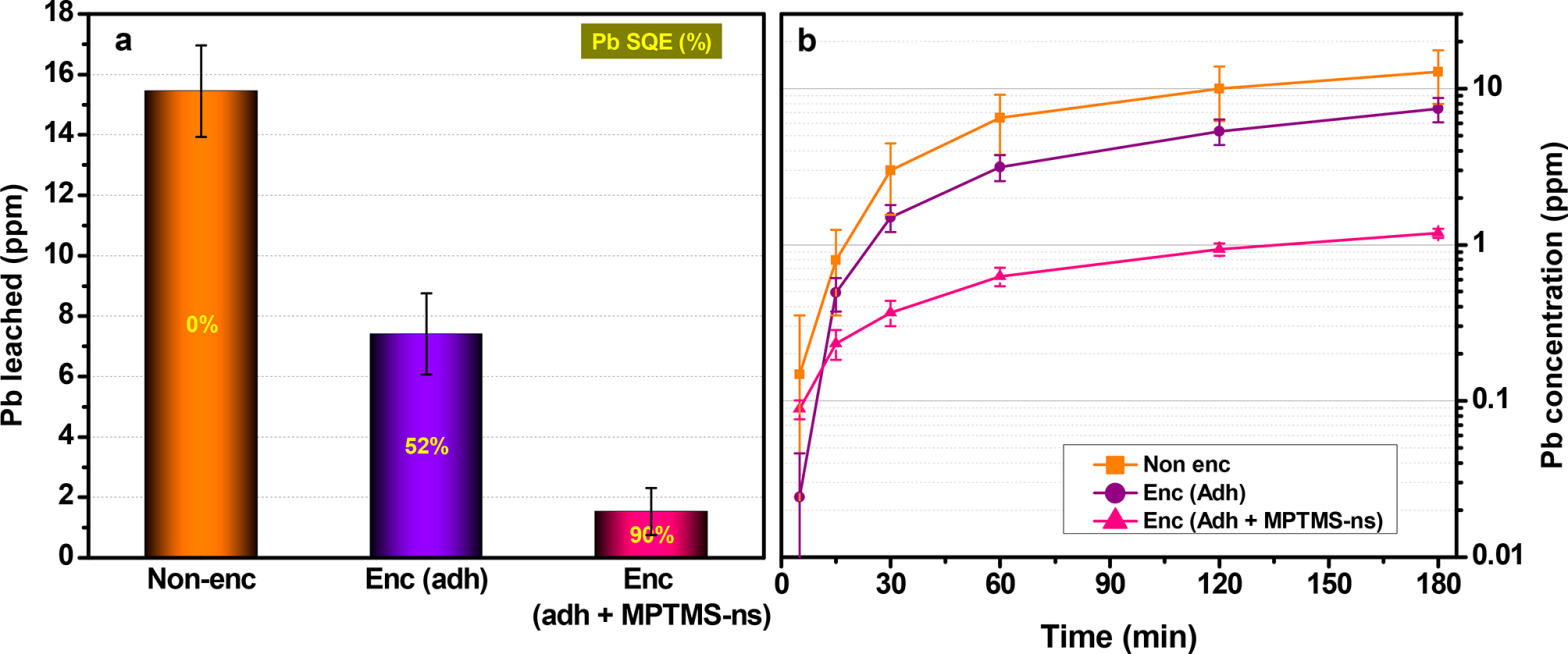
Average Pb SQE with MPTMS-ns. Pb^2+^-leached concentrations in water over 24 h (a) and during a span of 3 h (b) of nonencapsulated devices (orange), encapsulated devices with silicone-based adhesive only (purple), and encapsulated devices with the full sealant, i.e., adhesive + MPTMS-ns (pink).

We then used the encapsulant mixture for our PSCs as described in the Experimental Methods and illustrated in Figure 2. We dipped 12 nonencapsulated devices with 3.36 cm^2^ HaP area in 15 mL of water and measured an average Pb concentration of 15.26 ppm per device with FAAS. Furthermore, we encapsulated 4 PSCs of the same area and 15 glass substrates covered with HaP layers (not devices) with only the silicone-based adhesive and 28 PSCs with a mixture of the adhesive with MPTMS-ns. We characterized their *I*–*V* curves, then intentionally broke them, and conducted the same water dipping experiments as those with nonencapsulated devices to assess their Pb SQE. Our results suggest that our encapsulation with MPTMS-ns retains significantly more Pb^2+^ than the silicone-based adhesive by itself and can collect up to 90% of the otherwise leached Pb ions from broken devices (Figure 3a) during a 24 h span. The Pb leakage rate was also investigated by dipping encapsulated and nonencapsulated devices into water and sampling aliquots of the polluted water (Figure 3b) at different time lapses. The results indicate that most of the Pb leakage occurs within the first 2 h, and the rise of the Pb concentration for encapsulated devices with MPTMS-ns is 1 order of magnitude smaller than that for nonencapsulated devices. Encapsulation with the adhesive only (without MPTMS-ns) prevented dissolution of about half of the Pb apparently by hindering water permeation and transport into the HaP layer rather than by providing a chemical mechanism that would bind with the Pb. Scanning electron microscopy/energy-dispersive X-ray spectroscopy (SEM/EDS) was used to observe Pb retention on the broken devices. The EDS mapping results showed that most of the Pb remained embedded within the encapsulant surface in its PbI_2_ form (Figure S2). The PbI_2_ aggregates were observed by optical microscopy as well (Figure S3). The silicone adhesive is not completely impermeable because it allows for some water penetration when the encapsulated HaP is submerged in water for long periods of time, but the addition of MPMTS-ns does not affect the sealing quality negatively, as can be seen in the leaching tests in Figure S4; in fact, it seems to help to avoid water permeability, but further controlled reliability tests such as humid storing conditions (85% relative humidity) will be necessary to evaluate the water penetration. The transmittance spectra of encapsulated fluorine-doped tin oxide (FTO)/glass with/without MPTMS-ns can be seen in Figure S5, after the encapsulated cells were exposed to water. No difference in transmittance is seen between the samples with and without the nanospheres. We note that the PSC is illuminated through the FTO side of the cell and not through the top cover glass; hence, the lower transmittance of the cover glass (due to the encapsulant) does not reduce the light intensity that reaches the HaP absorber in the PSC.

### EPA Pb Limits in Water and Soil Compliance with Encapsulated PSCs

Within a 600-nm-thick layer of the total device, with 3.36 cm^2^ area, there should be nominally (for 100% dense layer) 275 μg of Pb (see the SI for calculations), which is close to the 231 μg (15.45 ppm) of Pb that we could collect from nonencapsulated devices. Because the EPA limit of Pb in drinkable water is 15 μg L^–1^, each nonencapsulated device can pollute up to 15 L of water if such were available and accessible underneath the cell. With the MPTMS-ns–silicone encapsulant, only 2 L of water will be polluted up to 15 μg L^–1^. A 1 m^2^ perovskite module with theMPTMS-ns–silicone encapsulant could leach up to 68 mg of Pb into the environment. For the soil underneath it, considering the top soil as the first 1 cm in depth and an average agricultural top-soil density^25^ of 13 kg m^–2^, we estimate to reach a 6.5 ppm Pb concentration in the top soil, which should be compared with 63 ppm from an nonencapsulated module (see the SI for calculations). The maximum EPA-allowed Pb concentration in agricultural soil is 200 ppm; the natural Pb occurrence will range from 10 to 100 ppm, depending on how intense human activity is/was in the region around the field.^5^

### PV Performance of Encapsulated Cells Is Not Affected by the Presence of MPTMS-ns

**Figure 4:**
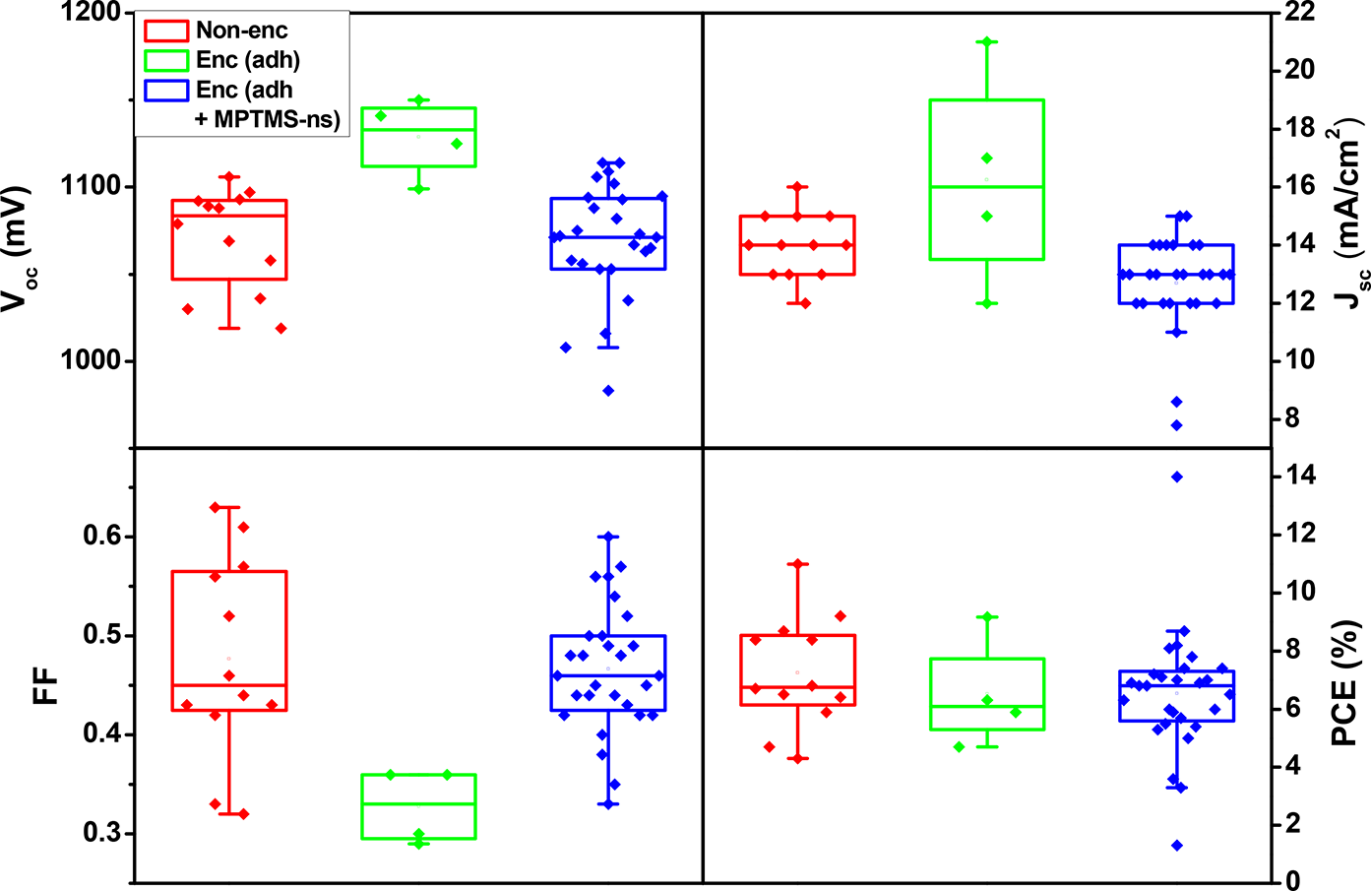
PV output statistics of 12 nonencapsulated PSCs (red), 4 encapsulated devices with only silicone adhesive (green), and 28 (adhesive + MPTMS-ns)-encapsulated PSCs (blue) from 4 different batches. Each dot represents an individual device. The hollow central dot represents the mean, the crossing bar inside the box represents the median, the lower and upper limits of the boxes are determined by 25% and 75% values, and the whiskers represent 95% and 5% values.

One of the most important aspects is that the encapsulant should not decompose the HaP layer and must trap the Pb as soon as the device breaks and gets dissolved with water, in either liquid or vapor form. By comparing the PV performance of encapsulated cells with nonencapsulated ones, we can address whether there is detrimental action of the encapsulant mixture. Device statistics (open-circuit voltage *V*_oc_, short-circuit current *J*_sc_, fill factor FF, and PCE) are shown in Figure 4 (from devices of four different batches: 12 nonencapsulated, 4 encapsulated with silicone-adhesive only, and 28 encapsulated with MPTMS-ns–silicone). Our main PCE (∼7%) and FF (∼0.48) values are low because of the large active areas (1 cm^2^), which translate into a higher probability of interfacial defects than with smaller device areas (0.038 cm^2^) with the same n–i–p architecture; such smaller cells had PCE 15.5%, FF 69%, *V*_oc_ 1.16 V, and *J*_sc_ 19.2 mA cm^–2^ (Figure S1). No significant difference between our encapsulated and nonencapsulated devices was observed, showing that there is no detrimental effect of the sealant on the device.

### MPTMS-ns Upscaling and Integration Costs Can Represent from 2.5% to 0.16% of PSC Production

The estimated cost of our thiol-functionalized nanoparticle sequestrant material is around ∼US$0.34 g^–1^ at the time of this publication (Table 1). Considering a 1 m^2^ PSC that contains 820 mg of Pb, we add 4 times that mass of MPTMS-ns (3.28 g). The costs of the other components [sodium dodecylbenzenesulfonate (SDBS) and HNO_3_] for nanosphere formation are negligible because they add no more than 1 cent. The total cost of the sequestering material is estimated to be 1.1 US$ m^–2^. This cost is based on prices for laboratory chemicals, which is only a small fraction of the total PV module cost (2.5% for today’s Si solar panels cost per square meter) and can be several times lower for industrial quantities. A report^22^ on silica coated by MPTMS produced in bulk quantities estimates 22 US$ kg^–1^, which reduces the price to 0.07 US$ kg^–1^.

**Table 1:** Price of MPTMS in Laboratory-Scale Quantities

material name	cost (US$)	vendor	package content (g)	unit cost per gram (US$)
MPTMS	171	TCI	500	0.34

For comparison, a thiol-containing hole-transport layer (PFDT)^17^ that was demonstrated to trap up to 84% of the Pb has a present consumer price of 110 USD g^–1^. Furthermore, its use requires the formation of a self-assembled monolayer on the HaP film, making it less practical for upscaling to a future perovskite PV industry. Cost estimates for other approaches are US$0.625 g^–1^ (phosphonic acid, EDTMP)^7^ and US$0.24 g^–1^ (CER),^9^ while uncommon sequestrants (such as DMPD)^7^ are more expensive. Even when the cost of CER is cheaper per gram, in practice, the relevant cost is per square meter, which depends on the mass of the encapsulant used and was not reported for any of the other approaches. According to the parameters for CER, we estimate a cost of US$3 m^–2^ (12.36 g m^–2^, total applied CER mass). In contrast, on the basis of our sequestrant and the methodology of this work, our estimated price per area of ∼US$1.1 /m^–2^ would add ∼2.5% to the cost of today’s commercial c-Si panels on a large scale (∼US$ 43 m^–2^)^21^ and is half the price of the EVA films used for encapsulation ($2 m^–2^, double layer). We note that this cost can be reduced further because of the price of MPTMS is much lower for bulk orders; it is estimated^22^ at ∼US$0.07 m^–2^ (0.16% of the cost of c-Si panels).

## Conclusions

The use of thiol-functionalized, silica core nanospheres (MPTMS-ns) in compatible encapsulants can prevent >90% of Pb leaching from PSC in the case of dissolution in water, reducing top-soil pollution to natural Pb levels (depending on whether the area is urban or rural and on other geographic and historical use factors), and drastically reducing the probability of water body pollution, especially of flowing water bodies. There is no significant difference in the PV performance of our cells with or without encapsulants. MPTMS-ns present an attractive and relatively low-cost route (2.5% of current silicon (c-Si) solar panel manufacturing costs, before cost reduction of MPTMS-ns by economy of scale) for incorporation into adhesives/encapsulants to achieve both Pb trapping properties and protection against ambient for PSCs.

## Experimental Methods

### Materials

All solvents and chemicals were used as received from the suppliers. Formadiminium iodide and methylammonium bromide were purchased from Greatcell Solar. Lead(II) iodide (PbI_2_) and lead(II) bromide (PbBr_2_) were purchased from TCI. 2,2′,7,7′-Tetrakis[*N*,*N*-bis(4-methoxyphenyl)amino]-9,9′-spirobifluorene (spiro-OMeTAD) was purchased from 1-Material Inc. *tert*-Butylpyridine, bis(trifluoromethanesulfonyl)imide lithium salt, cesium iodide (CsI), (3-mercaptopropyl)trimethoxysilane (MPTMS), and sodium dodecylbenzenesulfonate (SDBS) were purchased from Sigma-Aldrich. Tin(IV) oxide (SnO_2_) nanoparticles and Zn powder were purchased from Alfa Aesar. The fluorine-doped tin oxide (FTO)-coated glass substrates (<15 Ω per square) were obtained from Nippon Sheet Glass. The silicone-based sealant was purchased from Dow Corning.

### Device Fabrication

Devices were prepared on conductive FTO glass substrates. First, the device area was cut to a 2.4 cm × 2.4 cm area (5.76 cm^2^), and then FTO was etched away in a strip of 5 mm × 24 mm on the edge of the substrate by spreading Zn powder on it and adding 6 N HCl. Then, the substrates were cleaned thoroughly with dish soap, acid rinse, Decon 90 soap (at 70 °C), acetone, and isopropyl alcohol for 15 min each in an ultrasonic bath, alternating with a deionized water rinse between each step. The substrates were treated with O_2_ plasma for 4 min. A thin layer (∼30 nm) of SnO_2_ nanoparticles (7.5% in water) was deposited on the substrates by spin coating 70 μL of the solution on the substrate at 3000 rpm for 30 s. The substrates were annealed at 180 °C on a hot plate for 1 h. The triple-cation perovskite Cs_0.05_(MA_0.17_FA_0.83_)_0.95_Pb(I_0.83_Br_0.17_)_3_ and spiro-OMeTAD were prepared and coated as described in the literature.^26^ Two strips of HaP + spiro-OMeTAD of 5 mm × 24 mm each on the substrate edges were scratched off the cell with a blade, corresponding to the nonencapsulated area where the electrodes were deposited. Gold was thermally evaporated using a mask with a layout as described in Figure 2.

### PSC Encapsulation

MPTMS-capped nanospheres (MPTMS-ns) were synthesized as follows: MPTMS (3.724 g) and sodium dodecylbenzenesulfonate (SDBS; 0.0013 g) were added to water (30 mL) under vigorous stirring until the MPTMS droplets disappeared. NH_3_·H_2_O (0.5 mL) was added dropwise to the emulsion (pH 11.5), and the reaction mixture was held at 50 °C for 48 h. The colloidal dispersion was centrifuged, and the precipitate was washed three times with water, dried, and liophilized.^23^ MPTMS-ns was characterized by FTIR (Nicolet iS10) and high-resolution SEM (FEI, Magellan 400L) at 4 kV and 0.4 nA. After the preliminary studies on Pb absorption with MPTMS-ns, the Dow Corning silicone-based sealant was mixed with MPTMS-ns in a 4% (w/w) ratio. Soda lime glass spheres of 21–23 μm diameter were also added as a spacer in the same ratio. Approximately 28 mg of the encapsulant mixture were added on a 1.4 cm × 2.4 cm cover glass and spread over the entire cover glass surface for each device. The cover glass with encapsulant was mechanically pressed against the full device and secured with binder clips. The encapsulant was thermally cured at 80 °C for 2 h, and the device PV performance was subsequently measured.

### PSC Characterization

The PV performance was measured under simulated AM 1.5G illumination with a power density of 100 mW cm^–2^ (Oriel Sol3A Class AAA solar simulator with a Newport power supply 69920) in open-air room temperature conditions. Prior to each device batch measurement, the power output was recalibrated with a standard Si photodiode. The *J*–*V* characteristic curves were tested using a potentiostat (μAutolab Type II). A 1 cm^2^ shadow mask was employed to illuminate only the active area. Voltage scanning was applied in a backward and forward direction with a bias step fixed at 5 mV and a delay time at 0.05 s.

### Pb Absorption Measurements

FAAS with AAnalyst 400 (PerkinElmer) equipment with a Pb hollow-cathode lamp as the radiation source and a resonance line wavelength of 217 nm was used to determine the Pb concentration in solutions. The air/acetylene flame operated with a fuel rate of 3.14 L min^–1^, a lamp current of 12 mA, and a slit width of 0.7 nm. A calibration curve based on PbNO_3_ (2% HNO_3_) standard solutions (PerkinElmer) with a 0.9999 correlation coefficient was established as a reference to calculate the Pb content in polluted water solutions.

## Supporting Information Available

Additional experiments, soil pollution calculations, and cost estimates (PDF)
