# Supplementary material for: Lead Sequestration from Halide Perovskite Solar Cells with a Low-Cost Thiol-Containing Encapsulant
Source: ACS Appl Mater Interfaces. 2022 Jun 23;14(26):29766–72. doi: 10.1021/acsami.2c05074 (PMC9264311; doi:10.1021/acsami.2c05074)
Supplement: Supplementary file 1 — am2c05074_si_001.pdf [file am2c05074_si_001.pdf]

SUPPORTING INFORMATION

Pb sequestration from Halide Perovskite Solar Cells with a low-cost thiol-containing encapsulant

Rene D. Mendez L.[±], *Barry N. Breen*[§], *David Cahen*[±, °] *

±Dept. of Chemistry and Nanotechnology & Adv. Materials Center, Bar Ilan Univ., Ramat Gan 52900, Israel

§3GSolar Photovoltaics Ltd., Jerusalem 9777403, Israel.

°Weizmann Inst. of Science, Rehovot 76100, Israel.

AUTHOR INFORMATION

Corresponding Author * david.cahen@weizmann.ac.il

Calculations on Pb content in 1 m² PSC

For mixed perovskite composition such as $(CsI)_{0.05}(FAPbI_3)_{0.85}(MAPbBr_3)_{0.15}$, the mass percentage of Pb is 33.7% (207/614 g/mol) and the density is 4.12 g/cm^3[(1)]. Thus, the mass of Pb per unit area is about 1.38 g/m^2 for a 1 µm thick 100% dense film. For a typical thickness of 600 nm, the Pb content is 0.82 g/m^2. For 1 cm^2, which is the active area of our devices, there is an estimated content of 82 µg, while the entire surface area covered with perovskite in this study is 3.36 cm^2, which corresponds for a 600 nm thick film to 275 µg of Pb**.** In actual measurements, our non-encapsulated devices (3.36cm^2) leached ~229 µg of Pb which corresponds to 0.68 g of Pb in a 1 m^2 module.

The agricultural soil bulk density is ~1.3 g/cm^3 on average according to the US Dept. of Agriculture[2] . If we consider topsoil to be ~ 1 cm thick, the mass of agricultural topsoil is 13 kg/m^2. If all the Pb content from a 1 m^2 PSC leaches out and remains restricted to only that 1 m^2 area, the Pb concentration in the topsoil (D) would be:

$$D = \frac{B}{S}$$ (where *B* = Pb content in 1m^2 PSC, *S* = topsoil mass = 13 kg/m^2),

which yields 63 ppm for a non-encapsulated module and 6.5 ppm for modules with the MPTMS-ns-containing encapsulant.

I-V curve of a typical active area of 0.036 cm² PSC with n-i-p architecture.

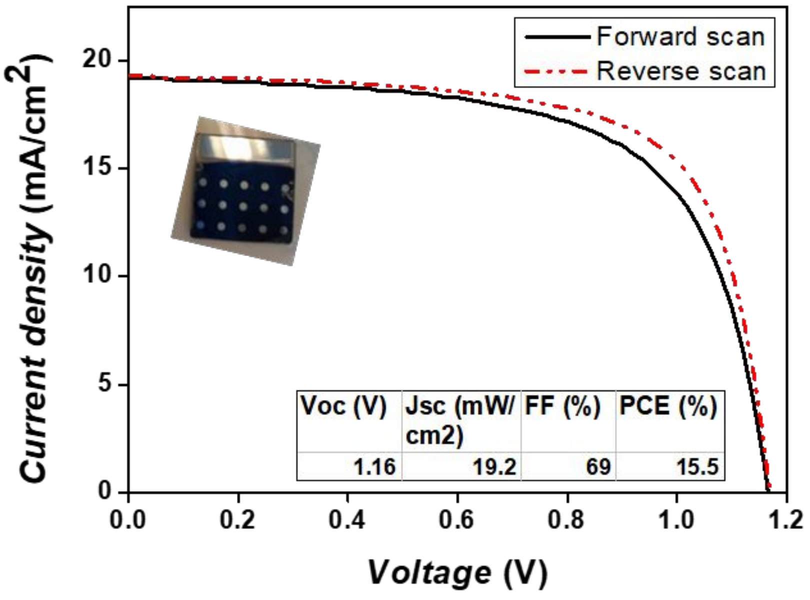

Figure.S1. *Typical I-V curve of an n-i-p PSC with 0.036 cm² active area, prepared with the same methodology as in the main text.* ***Inset****: top view of the PSC with the small top electrodes, showing that encapsulation would not allow making contact to the top electrodes (the dots).*

SEM/EDS and optical microscopy micrographs of remaining Pb in broken PSCs

The fragments of the broken encapsulated PSCs that had been exposed to water, were collected and analyzed by Scanning Electron Microscopy (SEM). PbI_2 agglomerates were found,

embedded within the encapsulant after exposure with water. Energy Dispersive X-Ray Spectroscopy (EDS) was employed to analyze the elemental composition of the sample.

Figure S2 shows the SEM (secondary electron) image and the EDS mapping of the relevant elements. Colored/white dots indicate the presence of the corresponding elements.

Figure S3 shows an optical microscopy image of the broken encapsulated PSC after exposure to water. While the MPTMS-ns were too small to be resolved by at 60x magnification, PbI_2 agglomerates were visible throughout the encapsulant surface (yellow color). The soda lime glass that is indicated was used as a spacer between the top cover glass and the PSC active layers, ensuring homogenous distribution of the silicone-adhesive throughout the whole area (as explained in the experimental methods section, PSC encapsulation, in the main text).

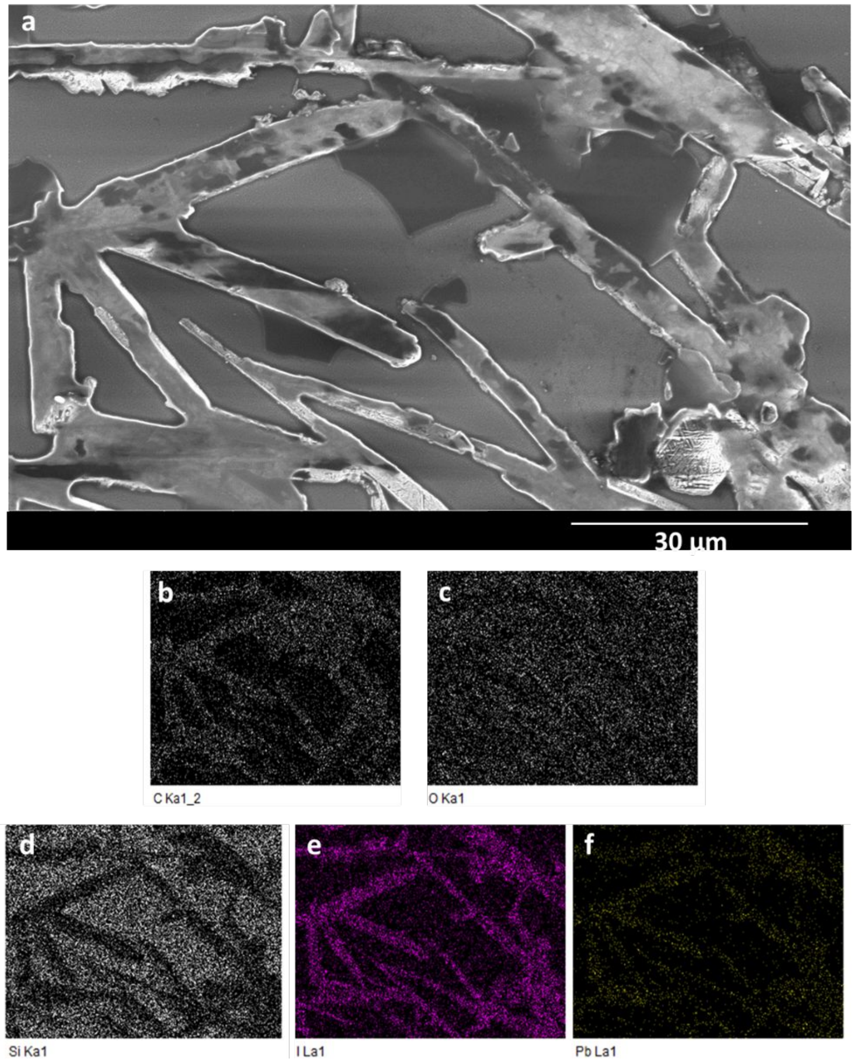

Figure S2. *SEM micrograph (Top left) and EDS mapping (within the same sample) of Carbon (b), Oxygen (c), Silicon (d), Iodine (e) and Lead (Pb) (f) in the PbI2 that remains on the encapsulant surface of broken PSCs.*

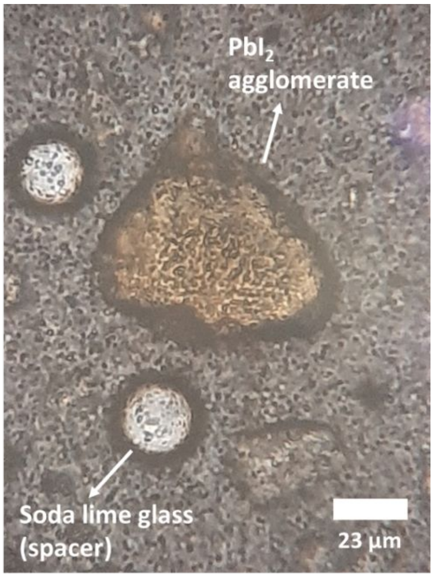

Figure S3. *Optical micrograph (60x) of PbI2 agglomeration in encapsulant of Si-adhesive + MPMTS-ns. The soda-lime glass spacer ensured homogeneous height in the adhesive coating between the top cover glass and the device.*

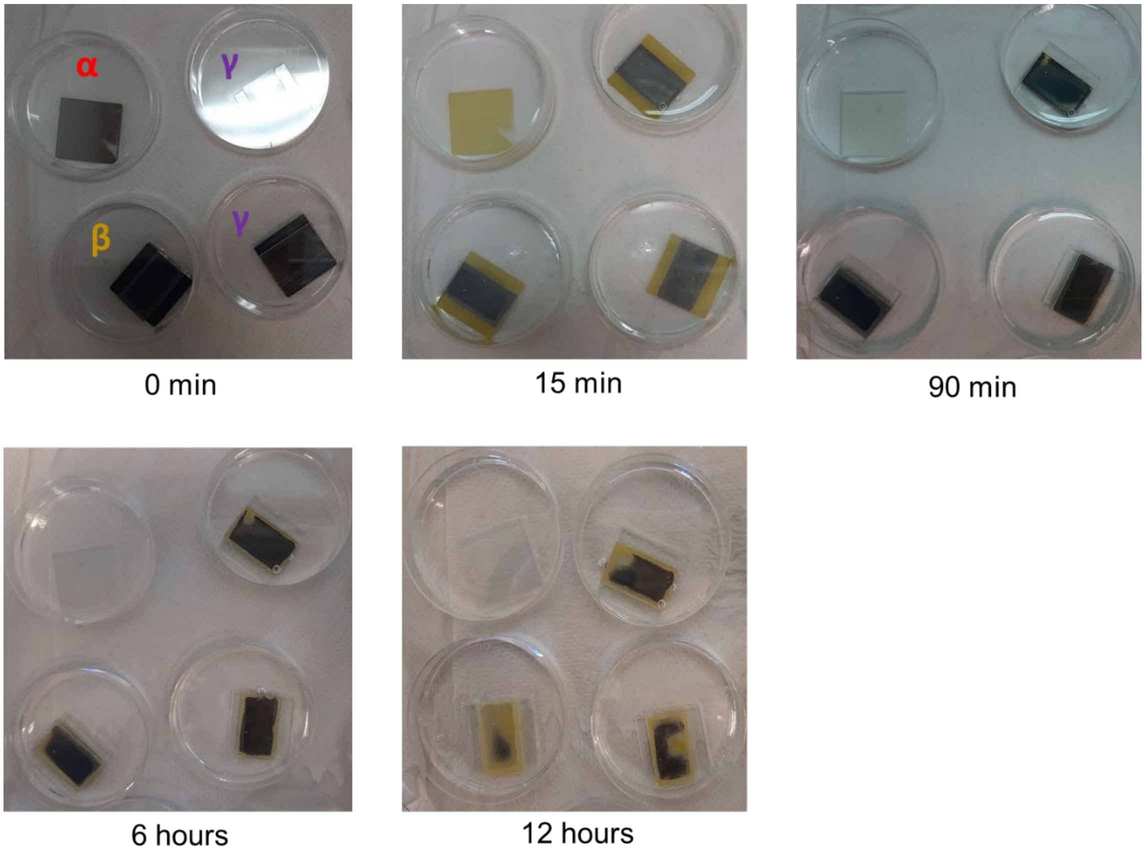

Figure S4. *Effect of time-dependent water diffusion in submerged edge-encapsulated HaP layers on glass substrates.* ***α*** *= non-encapsulated,* ***β*** *= encapsulated with silicone-adhesive only,* ***γ*** *=*

encapsulated with silicone-adhesive + MPTMS-ns. Sealing efficacy was, if anything, improved after including MPTMS-ns in the sealant.

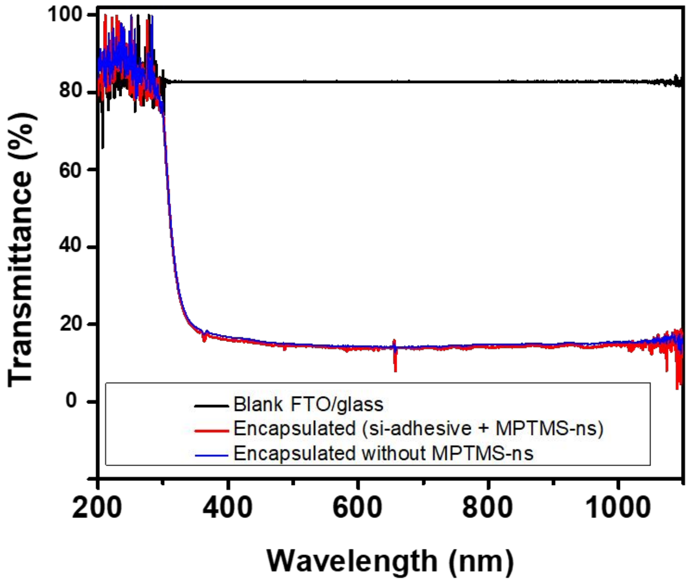

Figure S5. *UV/vis transmittance of FTO/glass used as cover glass before and after encapsulation with and without MPTMS-ns. The encapsulated glass/FTO samples were measured after being part of a PSC that was mechanically broken, and exposed to water.*

References

(1) Stoumpos, C. C.; Malliakas, C. D.; Kanatzidis, M. G. Semiconducting Tin and Lead Iodide Perovskites with Organic Cations: Phase Transitions, High Mobilities, and near-Infrared Photoluminescent Properties. *Inorganic Chemistry* **2013**, *52* (15), 9019–9038. https://doi.org/10.1021/ic401215x.

(2) USDA. *Web Soil Survey*. Natural Resources Conservation Service, United States Department of Agriculture. (accessed 2022-02-22).
